# Foldcomp: a library and format for compressing and indexing large protein structure sets

**DOI:** 10.1093/bioinformatics/btad153

**Published:** 2023-03-24

**Authors:** Hyunbin Kim, Milot Mirdita, Martin Steinegger

**Affiliations:** Interdisciplinary Program in Bioinformatics, Seoul National University, Seoul 08826, South Korea; School of Biological Sciences, Seoul National University, Seoul 08826, South Korea; Interdisciplinary Program in Bioinformatics, Seoul National University, Seoul 08826, South Korea; School of Biological Sciences, Seoul National University, Seoul 08826, South Korea; Institute of Molecular Biology and Genetics, Seoul National University, Seoul 08826, South Korea; Artificial Intelligence Institute, Seoul National University, Seoul 08826, South Korea

## Abstract

**Summary:**

Highly accurate protein structure predictors have generated hundreds of millions of protein structures; these pose a challenge in terms of storage and processing. Here, we present Foldcomp, a novel lossy structure compression algorithm, and indexing system to address this challenge. By using a combination of internal and Cartesian coordinates and a bi-directional NeRF-based strategy, Foldcomp improves the compression ratio by a factor of three compared to the next best method. Its reconstruction error of 0.08 Å is comparable to the best lossy compressor. It is five times faster than the next fastest compressor and competes with the fastest decompressors. With its multi-threading implementation and a Python interface that allows for easy database downloads and efficient querying of protein structures by accession, Foldcomp is a powerful tool for managing and analysing large collections of protein structures.

**Availability and implementation:**

Foldcomp is a free open-source software (GPLv3) and available for Linux, macOS, and Windows at https://foldcomp.foldseek.com. Foldcomp provides the AlphaFold Swiss-Prot (2.9GB), TrEMBL (1.1TB), and ESMatlas HQ (114GB) database ready-for-download.

## 1 Introduction

Fast and highly accurate structure prediction methods, such as AlphaFold2 (Jumper et al. 2021) and ESMFold (Lin et al. 2023), have generated an avalanche of publicly available protein structures. The AlphaFold database (Varadi et al. 2021) and ESMatlas (Lin et al. 2023) contain over 214 million and 617 million predicted structures in PDB format, respectively. A compressed local copy would require 25 and 15 TB storage, respectively. These databases are biological treasure troves but analysing them is challenging due to these technical aspects.

The PDB or mmCIF (Westbrook et al. 2022) formats store protein structures as atom records in an 80-byte columnar plain-text format that includes the Cartesian coordinates. Various strategies (Valasatava et al. 2017) have been proposed to deal with the growth of protein structure databases, including general-purpose compressors like Gzip and data-record-specific encodings like BinaryCIF (Sehnal et al. 2020) and MMTF (Bradley et al. 2017). PIC (Staniscia and Yu 2022) transforms 3D coordinates into a lossy 2D image-like format and applies the PNG-image compression algorithm. Specialized formats for molecular trajectories (Roe and Brooks 2022) have also been developed to compress different states of a same molecule.

Here, we present Foldcomp, a software and library that implements a novel algorithm to compress PDB/mmCIF using anchored internal coordinates combined with an indexing strategy to store large structural sets. We provide a command-line interface, a library/API for inclusion in other projects, and a Python interface to (de)compress and efficiently load user-selected entries in sequential- or random-access order.

## 2 Materials and methods

**Figure 1.**
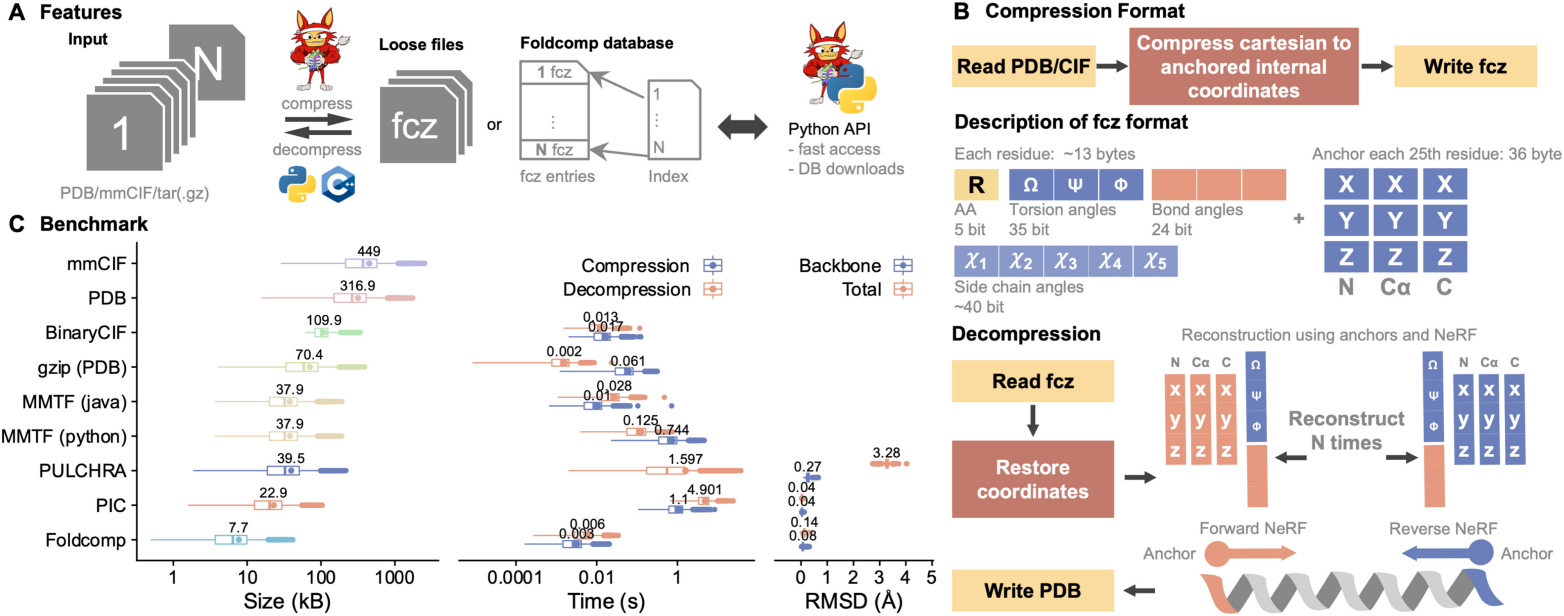
(A) Foldcomp is a library to compress, store and index protein structures. Foldcomp is written in C++ and comes with a command line and Python interface to compress, decompress, and access structures. (B) *Compression* takes 3D atom coordinates stored in PDB/mmCIF format as input and calculates and stores all internal coordinates, backbone torsions, and bond angles, and additionally, for every Nth residue (by default every 25th) the 3D atom coordinates for the N, C, and C-alpha atoms as anchor coordinates in its fcz format. By using anchors, we can prevent the accumulation of decoding errors. *Decompression* uses the anchor coordinates and internal coordinates to reconstruct the 3D atom coordinates by first extending the coordinates from N-terminal to C-terminal (forward) and then from C-terminal to N-terminal (backward) using Natural Extension Reference Frames (NeRF; Parsons et al. 2005), followed by averaging the coordinates in between. Averaging reduces the reconstruction error by approximately a factor of two. (C) Comparison of file size (left), compression/decompression speed (middle), and backbone/all-atoms reconstruction error (right) for lossless and lossy protein structure compressors using the *Saccharomyces cerevisiae* proteome from the AlphaFold DB

Foldcomp’s workflow and file format is illustrated in Fig. 1A and B.

### 2.1 Input

Foldcomp compresses PDB/mmCIF files stored in various input formats, such as individual-, directories of-, or optionally compressed tar-archives of PDB/mmCIF files. It returns compressed binary files (fcz format) in an individual directory, tar-archive, or Foldcomp database.

### 2.2 Index

All compressed entries are concatenated and stored in a single file. We keep track of the entry identifier, start position, and length in separate plain-text files. This format is compatible with the MMseqs2 (Steinegger and Söding 2017) database format, which was initially inspired by the ffindex database format (unpublished). We implemented support for Foldcomp databases in Foldseek (van Kempen et al. 2022).

### 2.3 Python

Foldcomp’s Python interface can be installed using pip install foldcomp. We provide the functionality to download prebuilt databases, compress and decompress individual files, and iterate.

## 3 Results

We compared Foldcomp to state-of-the-art software (see Fig. 1C) using the *Saccharomyces cerevisiae* proteome from the AlphaFold DB v4. Among the compressors tested [PIC, PULCHRA (Rotkiewicz and Skolnick, 2008), MMTF-python, Ciftools-java, and Gzip; see Supplementary Materials], Foldcomp was the most efficient in terms of speed and size. It required 0.003 and 0.006 s for compression and decompression, respectively, and had a size of 7.7 kb while maintaining one of the lowest reconstruction errors of 0.08 Å and 0.14 Å for backbone and all-atoms among the lossy compressors. Using 16 threads reduced the time for compression and decompression to 1.617 and 2.532 s, respectively, resulting in a speed-up of 13× compared to single-core.

### 3.1 Databases

We provide a Foldcomp version of the AlphaFold database (v4) Swiss-Prot, TrEMBL, and ESMatlas high-quality requiring 2.9 GB, 1.1 TB, and 114 GB, respectively. This is an order of magnitude smaller than the original size. Our databases are hosted on CloudFlare R2 for fast downloads and can be easily accessed through the Python interface.

## 4 Limitations

Currently, Foldcomp only supports single-chained protein structures without missing residues. We plan to extend the format to deal with discontinuities and multiple chains in the future. Foldcomp is not meant to replace the PDB/mmCIF format, since these contain valuable meta-information that is discarded by Foldcomp.

## 5 Conclusion

Foldcomp’s high speed combined with its novel algorithm to efficiently compress, and index structures will enable researchers to easily explore large collections of predicted protein structures on consumer hardware. We anticipate that easy access to billions of predicted protein structures will advance the field of protein structure analysis.

## Supplementary Material

btad153_Supplementary_Data

## Data Availability

The data used for benchmarking is available in the AlphaFold database, at https://ftp.ebi.ac.uk/pub/databases/alphafold/v4/UP000002311_559292_YEAST_v4.tar. Foldcomp-compressed databases of AlphaFold database and ESMatlas are available at https://foldcomp.steineggerlab.workers.dev/.
