# Supplementary material for: Foldcomp: a library and format for compressing and indexing large protein structure sets
Source: Bioinformatics. 2023 Mar 24;39(4):btad153. doi: 10.1093/bioinformatics/btad153 (PMC10085514; doi:10.1093/bioinformatics/btad153)
Supplement: btad153_Supplementary_Data [file btad153_supplementary_data.zip › supplementary_document.pdf]

Supplementary Material for Foldcomp: a library and format for compressing and indexing large protein structure sets

Hyunbin Kim,[1] Milot Mirdita,[2] and Martin Steinegger[1,2,3,4]

[1] *Interdisciplinary Program in Bioinformatics, Seoul National University, Seoul, South Korea.*
[2] *School of Biological Sciences, Seoul National University, Seoul, South Korea*
[3] *Institute of Molecular Biology and Genetics, Seoul National University, Seoul, South Korea*
[4] *Artificial Intelligence Institute, Seoul National University, Seoul, South Korea*

WORKFLOW

Input Foldcomp is designed to handle various types of inputs, including individual protein structure files in PDB or mmCIF format, as well as directories containing multiple structures, (gzipped) tarball archives, and Foldcomp databases. Foldcomp uses structure reading modules from gemmi [1] and a modified version of microtar for tarball processing. All file processing can be parallelized using OpenMP.

FCZ file format To facilitate efficient storage of angles and coordinates, we have developed a novel binary file format named FCZ. The FCZ format comprises four distinct components: header, backbone, side-chain, and b-factor. The header contains information regarding the starting coordinates, parameters for discretizing angles, and the starting index and size of indices. The backbone of a single amino acid is efficiently represented using 8 bytes, as described in detail in **Table S1**. Additionally, the absolute coordinates of the backbone of the anchor residue are also included in the backbone component. The torsion angles in side-chains and b-factors are compactly encoded using 1 byte, with a minimum value represented as 0 and a maximum value represented as 255. For most predicted structures, which are the primary use case for Foldcomp, b-factors are per-residue confidence scores, and thus we store a single b-factor per amino acid. During the compression step, the inner coordinates, including torsion angles, are determined and integrated into the FCZ file, enabling the reconstruction of the original coordinates upon decompression.

Decompression The reconstruction of cartesian coordinates from inner coordinates was carried out using the Natural Extension Reference Frame (NeRF) algorithm. The NeRF algorithm requires three initial cartesian coordinates, the bond length, the bond angle, and the torsion angle. The coordinates of subsequent atoms were calculated iteratively from the inner coordinates stored in the FCZ file. We used constant values for bond lengths and encoded angles in a fixed number of bits. However, errors may arise due to the decoding of angles from discretized integers, leading to deviations from the original coordinates as the input peptide grows longer. To mitigate error accumulation, we employed a bi-directional approach in which the NeRF algorithm was run twice in opposite directions and the coordinates were averaged from both results. To further reduce the deviation, we weighted the coordinates based on the given atom's distance from the starting point. The bi-directional NeRF approach with weighted averaging reduced the deviation by half and, combined with internal anchor points, resulted in a total RMSD reduction to within 0.08 Å.

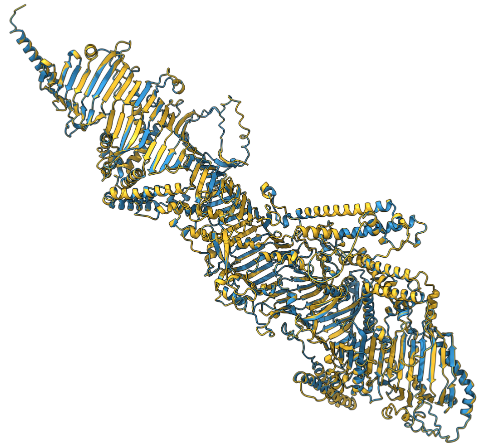

FIG. S1: FMP27 (Q06179) is a mitochondrial protein with 2,628 amino-acids and 21,382 atoms, and also the largest protein in the AlphaFoldDB Yeast dataset. Saving this structure in fcz format requires 41.6 KB instead of 1.75 MB (PDB) with backbone RMSD of 0.079Å, and all-atoms RMSD of 0.14Å.

Database The Foldcomp database utilizes the MMseqs2 database format to mitigate the overhead associated with storing a large number of small files in a file system. The database format consists of three files, namely the data file itself, the ".index" file, and the ".lookup" file. The data file is a concatenated data file, with each individual file separated by a null byte. This design results in a reduction of per-file padding compared to tarball archives, where each entry and its header are padded to 512-byte boundaries, and reduces up to 12% storage requirements for compressing the AlphaFold SwissProt database. The ".index" file contains the internal numeric identifier, offset, and size of each file, which can be searched through binary search. The ".lookup" file maps the internal numeric identifier to the file name. With this format, the database is optimized for efficiently iterating through large datasets, and is accessible through a Python API.

BENCHMARK

To benchmark protein structure compression tools, we used predicted structures of *Saccharomyces cerevisiae* proteome from AlphaFold database version 4 available at https://ftp.ebi.ac.uk/pub/databases/alphafold/latest/UP000002311_559292_YEAST_v4.tar **Table S2** shows the databases we used in this paper. The benchmark tests were repeated five times and we averaged run times over five trials. All runtimes were measured on a server with an AMD EPYC 7702P 64-Core CPU and 1TB RAM. All compression/decompression measurements are available in **Supplementary Data 1**. All benchmarks described were conducted on a RAM disk (/dev/shm) to minimize the effect of I/O operations for all the tools. **Table S3 and S4** show runtime measurements executed on an NVMe SSD and HDD. These show similar runtimes to the in-RAM runtimes except for higher sys time.

As Foldcomp supports batch mode for compression and decompression, we also used batch mode for the other compressors, if possible. For the tools that do not have a batch mode, we either implemented a batch mode or used GNU parallel (version 20220722) [2].

Foldcomp was run in batch mode, which is automatically activated if a directory with PDB/mmCIF files is given as an input (or activated if a tar file containing PDB/mmCIF files is given). For time measurements, we use the –time flag, which measures the runtime of functions for compression and decompression with std::chrono::high_resolution_clock. Utilizing a single thread for Foldcomp resulted in a maximum RAM usage of 21 megabytes (**Table S5**) and a compression and decompression time of 20 and 35 seconds respectively for the benchmark dataset. This performance can be improved through multithreading, as demonstrated by the reduction of the compression and decompression time to 1.5 seconds and 2.5 seconds when utilizing 16 threads. **Figure S1** shows the longest protein in AlphaFold-YEAST decompressed by Foldcomp, and superposed to the original PDB file using ChimeraX [3].

Gzip was executed using the recursive (-r) option for batch running and we added timestamps using ts from moreutils [4] to each entry to measure runtimes.

PIC was executed using the recursive (-r) option. To cut the total runtime, we split the input directory into the number of processes to run and applied parallel. We applied the -rk flag for compression and -rdk flag for decompression. During the batch execution loop, we measured the runtimes of PICCompress and PICDecompress with time.process_time.

CIFTools is written in Java and has APIs to read/write mmCIF and binary CIF formats. As it lacks a command line interface for batch execution, we wrote CompressBatch.java and DecompressBatch.java to benchmark. We measured the runtime of CifIO.readFromPath, and either CifIO.writeBinary (compression) or CifIO.writeText (decompression) with System.nanoTime.

MMTF has been integrated into BioJava [5] structure module and BioPython's [6] Bio.PDB API. Using the API, we wrote scripts for each implementation that can be run through the command line. MMTF supports lossy compression by saving coordinates with with limited precision using the given multiplier. However, by itself, the lossy MMTF format does not improve compression. It needs to additionally be compressed with e.g., gzip compression (**Supplementary Data 1**). On average, gzipping lossy MMTF with a multiplier of 10 saved 33.6% compared to gzipping lossless MMTF (avg. 16 KB vs. 24.8 KB file size), while maintaining an RMSD of 0.05 Å. We did not include gzipped lossy MMTF in the benchmark, since it would be the only tool that requires an additional compression step after compression.

MMTF-JAVA The PDB files were processed using the PDBFileReader and were converted into the MMTF format using the MmtfActions.writeToFile method. Decompression was performed using MmtfActions.readFromFile and Structure.toPDB.

MMTF-python In the compression step, mmCIF files were loaded with MMCIFParser and MMTF files were written with MMTFParser. MMTFIO and PDBIO were used to read MMTF files and write PDB output during decompression. We measured runtime with time.process_time.

PULCHRA reconstructs whole peptides from C-alpha atoms using an optimization procedure and was not originally developed as a compression tool. We can achieve size reduction of PDB files by discarding all non-C-alpha atoms. As this is not an actual compression procedure, we have omitted the compression time number. The compression runtime number in the raw data table is based on grep-ping C-alpha atoms in PDB files. We used the runtimes reported by Pulchra.

Summary **Tables S6 and S7** show a summary of the benchmark results. Foldcomp is the compression tool with the fastest compression and the second fastest decompression speed. The FCZ format has the highest compression ratio among all available structure compression tools, making it an efficient solution for structure data compression. Scripts used to benchmark are available at: https://github.com/steineggerlab/foldcomp-analysis. Used software versions for all tools are listed in **Table S8**, included libraries used to generate **Figure 1c**.

Header	Bits	Data types	Bits
Magic number	32 x 1	Residue	5
First index	16 x 2	Omega angle	11
Index size	16 x 2	Psi & Phi	12
Size of side-chains	32 x 1	BA	8
First residue	8 x 1	SC-TA	8
Starting atoms	32 x 3	B-factor	8
TA min & bin size	32 x 2 x 6	Anchor	32 x 3 x 3

TABLE S1: Number of bits used to encode data types in FCZ format. BA for bond angles, TA for torsion angles, and SC for side-chain.

Type	Threads	Max RAM (kb)
Compression	1	21132
	4	51056
	16	164168
Decompression	1	19944
	4	33416
	16	82200

TABLE S5: Maximum RAM usage of Foldcomp on compressing and decompressing AlphaFoldDB-YEAST dataset with different thread counts.

Database	Subset	Version	Entries	Size
AlphaFoldDB	YEAST	v4	6039	1.8 GB
AlphaFoldDB	TrEMBL	v4	214 M	23 TB
AlphaFoldDB	SwissProt	v3	542378	26 GB
ESMatlas	HQ	v0	37 M	4.3 TB

TABLE S2: Structure databases used in this manuscript.

Tool	Mean	Standard deviation	Minimum	Maximum
Foldcomp	0.003s	0.003s	0.0002s	0.021s
	0.006s	0.004s	0.0003s	0.034s
PIC	1.100s	0.746s	0.1058s	8.402s
	4.901s	2.044s	0.6622s	24.279s
Pulchra	1.597s	3.067s	0.0020s	37.724s
MMTF (python)	0.744s	0.580s	0.0226s	4.664s
	0.125s	0.097s	0.0039s	0.760s
MMTF (java)	0.010s	0.012s	0.0007s	0.722s
	0.028s	0.022s	0.0011s	0.476s
gzip	0.061s	0.045s	0.0012s	0.319s
	0.002s	0.001s	<0.0001s	0.023s
BinaryCIF	0.017s	0.012s	0.0020s	0.124s
	0.013s	0.009s	0.0015s	0.116s

TABLE S6: Speed benchmark results on compressing and decompressing AlphaFoldDB-YEAST. Compression results in row 1, decompression in row 2. Pulchra: decompression only.

Disk type	Threads	Time (real)	Time (sys)	Time per entry
RAM	1	19.417 s	0.560 s	0.0032 s
RAM	4	5.300 s	0.568 s	0.0009 s
RAM	16	1.617 s	0.603 s	0.0003 s
NVMe	1	19.668 s	0.624 s	0.0033 s
NVMe	4	5.063 s	0.708 s	0.0008 s
NVMe	16	1.418 s	0.930 s	0.0002 s
HDD	1	33.513 s	1.068 s	0.0055 s
HDD	4	8.042 s	1.111 s	0.0013 s
HDD	16	4.124 s	2.257 s	0.0007 s

TABLE S3: Benchmark of Foldcomp on compressing AlphaFoldDB-YEAST dataset with different thread counts and on different disk types.

Disk type	Threads	Time (real)	Time (sys)	Time per entry
RAM	1	34.405 s	0.796 s	0.0057 s
RAM	4	9.318 s	0.776 s	0.0015 s
RAM	16	2.532 s	34.323 s	0.0004 s
NVMe	1	35.394 s	1.647 s	0.0058 s
NVMe	4	9.221 s	2.313 s	0.0015 s
NVMe	16	2.454 s	2.218 s	0.0004 s
HDD	1	62.408 s	1.883 s	0.0103 s
HDD	4	15.001 s	2.036 s	0.0024 s
HDD	16	4.473 s	2.387 s	0.0007 s

TABLE S4: Benchmark of Foldcomp on decompressing AlphaFoldDB-YEAST dataset with different thread counts and on different disk types.

Tool	Mean (kb)	Standard deviation (kb)	Minimum (kb)	Maximum (kb)
Foldcomp	7.713	5.671	0.492	41.601
PIC	22.897	14.406	1.574	103.825
MMTF	37.884	25.474	3.620	191.323
Pulchra	39.498	29.507	1.863	216.999
gzip	70.444	52.424	4.004	388.984
BinaryCIF	109.907	38.406	61.184	339.917
PDB	316.854	237.453	15.551	1752.434
CIF	448.993	338.885	28.382	2539.558

TABLE S7: Compression size benchmark results on compressing and decompressing AlphaFoldDB-YEAST.

Name	Version	Comment
Foldcomp	Git: d9c08f5	Benchmarks
Foldcomp	Git: 4acf954	Compressing databases
PIC	Git: 1f80379	Benchmarks
PULCHRA	v3.0.4	Benchmarks
MMTF-python	v1.1.3	Benchmarks
MMTF-java	v1.0.11	Benchmarks
BioJava	v6.1.0	Benchmarks
CIFTools-java	v2.0.2	Benchmarks
Gzip	v1.6	Benchmarks
GNU parallel	v20220722	Multiprocessing
R [7]	v4.2.1	Visualization
ggplot2 [8]	v3.4.0	Visualization
cowplot [9]	v1.1.1	Visualization
ggpubr [10]	v0.5.0	Visualization
deeptime [11]	v0.3.0	Visualization
shadowtext [12]	v0.1.2	Visualization
reshape2 [13]	v1.4.4	Visualization
Python	3.8.15	Benchmark
OpenJDK	11.0.15	Benchmark
Micromamba	1.0.0	Benchmark
ChimeraX	1.4	Visualization

TABLE S8: Software versions used in this manuscript.

REFERENCES

[1] Wojdyr, M. Gemmi: A library for structural biology. *Journal of Open Source Software*, **7**(73), 4200, 2022.

[2] Tange, O. Gnu parallel 20220722 ('roe vs wade'), 2022. URL https://doi.org/10.5281/zenodo.6891516. GNU Parallel is a general parallelizer to run multiple serial command line programs in parallel without changing them.

[3] Pettersen, E.F. et al. UCSF ChimeraX: Structure visualization for researchers, educators, and developers. *Protein Sci.*, **30**, 70–82, 2021.

[4] Hess, J. moreutils, 2022. URL https://joeyh.name/code/moreutils. moreutils is a collection of the unix tools that nobody thought to write long ago when unix was young.

[5] Lafita, A. et al. Biojava 5: A community driven open-source bioinformatics library. *PLOS Computational Biology*, **15**(2), 1–8, 2019.

[6] Cock, P.J. et al. Biopython: freely available python tools for computational molecular biology and bioinformatics. *Bioinformatics*, **25**, 1422–1423, 2009.

[7] R Core Team. *R: A Language and Environment for Statistical Computing*. R Foundation for Statistical Computing, Vienna, Austria, 2022. URL https://www.R-project.org/.

[8] Wickham, H. *ggplot2: Elegant Graphics for Data Analysis*. Springer-Verlag New York, 2016. ISBN 978-3-319-24277-4. URL https://ggplot2.tidyverse.org.

[9] Wilke, C.O. *cowplot: Streamlined Plot Theme and Plot Annotations for 'ggplot2'*, 2020. URL https://CRAN.R-project.org/package=cowplot. R package version 1.1.1.

[10] Kassambara, A. *ggpubr: 'ggplot2' Based Publication Ready Plots*, 2022. URL https://CRAN.R-project.org/package=ggpubr. R package version 0.5.0.

[11] Gearty, W. *deeptime: Plotting Tools for Anyone Working in Deep Time*, 2022. URL https://CRAN.R-project.org/package=deeptime. R package version 0.3.0.

[12] Yu, G. *shadowtext: Shadow Text Grob and Layer*, 2022. URL https://CRAN.R-project.org/package=shadowtext. R package version 0.1.2.

[13] Wickham, H. Reshaping data with the reshape package. *J. Stat. Softw.*, **21**, 1–20, 2007.
