# Supplementary figures and images for: Foldcomp: a library and format for compressing and indexing large protein structure sets
Source: Bioinformatics. 2023 Mar 24;39(4):btad153. doi: 10.1093/bioinformatics/btad153 (PMC10085514; doi:10.1093/bioinformatics/btad153)

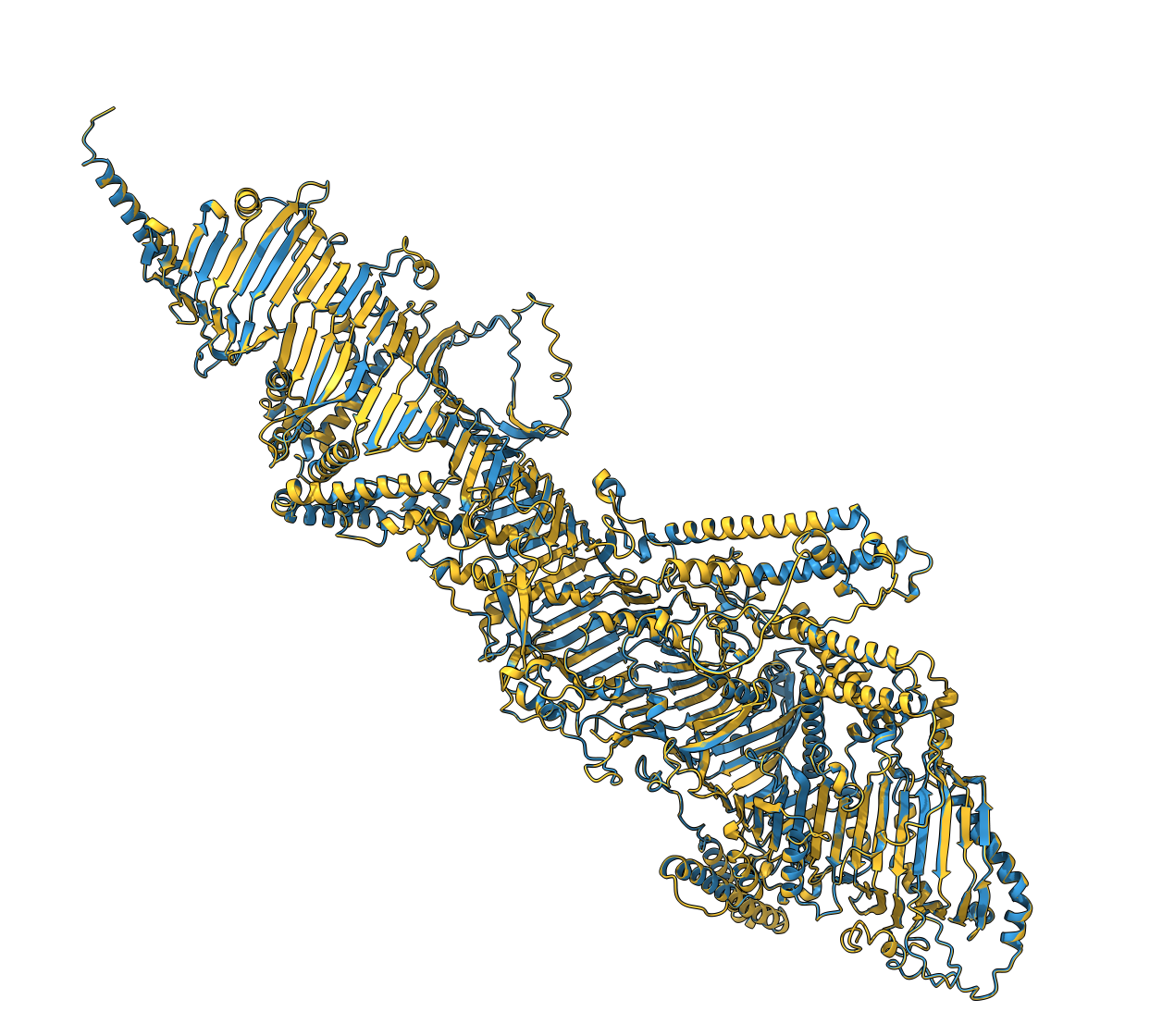

Supplement: btad153_Supplementary_Data [file btad153_supplementary_data.zip › supplementary_fig1.png]

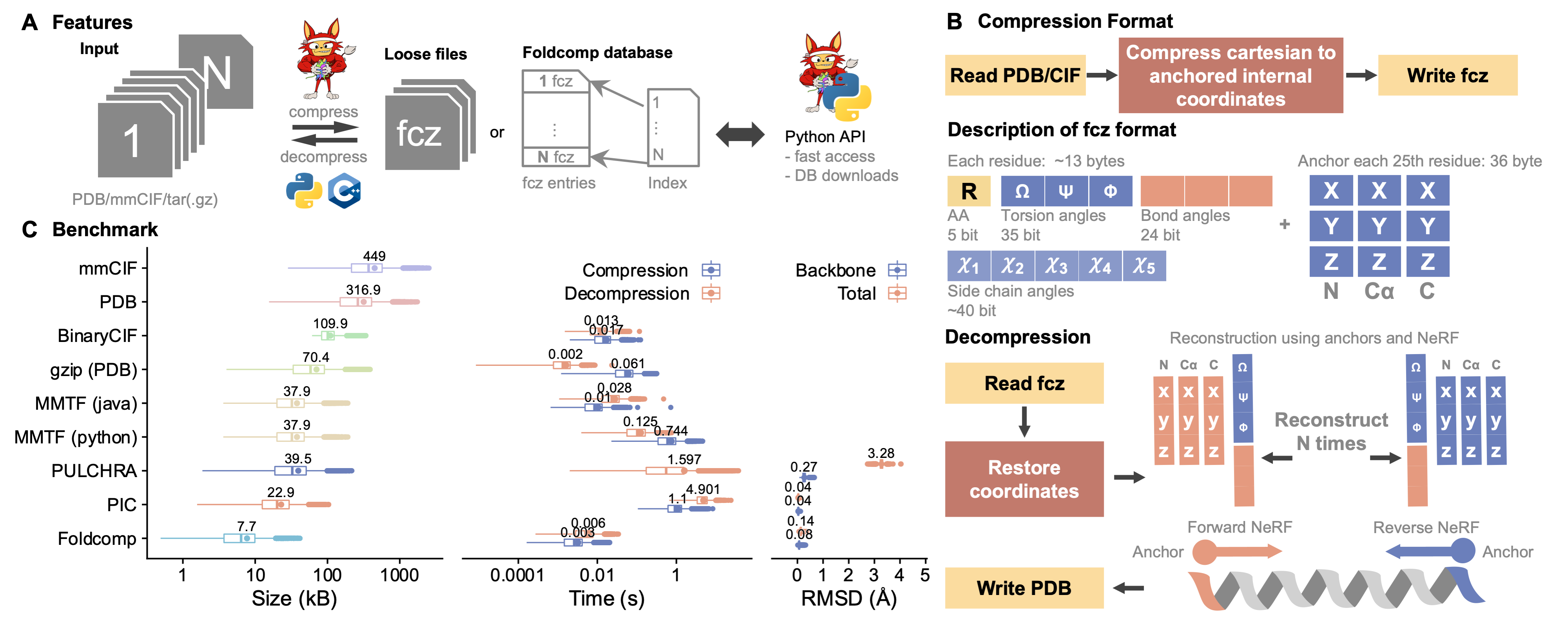

Supplement: btad153_Supplementary_Data [file btad153_supplementary_data.zip › main_fig1.tiff]
